# An evolutionary genomic perspective on preterm birth, genome editing, and pregnancy in the human species

**DOI:** 10.3389/fgeed.2026.1805932

**Published:** 2026-06-17

**Authors:** Akua A. Obeng, Monica Uddin, Chengqi Wang, Derek E. Wildman

**Affiliations:** Global, Environmental, and Genomic Health Sciences, College of Public Health, University of South Florida, Tampa, FL, United States

**Keywords:** comparative genomics, CRISPR, DOHAD, evolutionary genomics, gene editing, placental evolution, population genetics, preterm birth

## Abstract

The processes of labor and birth have a complex evolutionary history, with substantial variation among species showing differences in gestational length, offspring number, anatomy, and rates of fetal development. Understanding the genomic basis of pregnancy is therefore a focus of evolutionary research, given the importance of reproductive success in processes such as natural selection, mutation, genetic drift, and migration. Disruptions to normal pregnancy processes include preterm birth, which can arise from multiple factors, including infection, anatomical variation, injury, age, parity, and multiple gestation and other obstetrical syndromes as well (e.g., preeclampsia, and stillbirth). These factors each influence unique and overlapping networks of candidate genes and biological pathways. Here we synthesize evidence from comparative genomics, population genetics, and vertebrate reproductive biology to show that many PTB-relevant genes, including those involved in progesterone signaling, innate immunity, placental regulation, and chromosome 19 gene clusters, have undergone lineage- or population-specific evolutionary change. Integrating evolutionary insights with functional genomics, machine learning, and modern genome-editing technologies, we provide a principled framework to distinguish conserved, high-risk targets from evolutionarily flexible loci, guiding safer mechanistic studies and future interventions to reduce PTB risk. From an initial list of approximately 1,500 genes involved in pregnancy, we identified those that show evidence of recent evolutionary change for which functional inference is possible. We review some specific nucleotide sites that, when disrupted via CRISPR gene editing, are likely to impact the processes of labor and birth. These loci fall within protein coding genes, transposable elements, transcription factor binding sites, and non-coding RNAs. They are found in nuclear hormone receptors (e.g., *PGR*), genes with placenta- and uterine-specific expression patterns (e.g., *LGALS13*), as well as signaling molecules and immunological loci. Finally, we provide evidence that gene activity and sequence variation differ across species and provide examples of pathway differences between chimpanzees (nociception) and humans (inflammation).

## Introduction

Preterm birth (PTB), defined as delivery occurring before 37 completed weeks of gestation, remains a preventable global health challenge and one of the leading causes of neonatal morbidity and mortality (Cao et al., 2022). An estimated 13.4 million newborns were born preterm, accounting for 9.9% of global births in 2020 (Ohuma et al., 2023). There is considerable variation in PTB rates across regions. The highest prevalence was observed in Southern Asia, at 13.2 per 1,000 live births, nearly twice the lowest regional rate reported in Eastern and South-Eastern Asia and Oceania (6.8 per 1,000 live births), with sub-Saharan Africa following at 10.1 per 1,000 live births. Considerable heterogeneity was evident within regions. In Southern Asia, the highest national rates were reported in Bangladesh (16.2 per 1,000 live births), Pakistan (14.4 per 1,000 live births), and India (13.0 per 1,000 live births). In Latin America, rates ranged from 5.8 per 1,000 live births in Nicaragua to 12.8 per 1,000 live births in Suriname. Similarly, in sub-Saharan Africa, Ethiopia (12.9 per 1,000 live births) and the Democratic Republic of the Congo (12.4 per 1,000 live births) reported among the highest national rates. (Ohuma et al., 2023). In the United States, the preterm birth rate in 2023 was 10.41% of live births and was unchanged from 2022 (Osterman et al., 2023).

Preterm birth (PTB) is associated with adverse neonatal outcomes related to organ immaturity and long-term complications, including vision and hearing impairments (Crump, 2020). Neonatal survival after preterm birth varies widely worldwide, with about 69% of extremely preterm infants (<28 weeks gestation) surviving in high-income countries while fewer than 32% surviving in low-income countries (Getaneh et al., 2025). Preterm infants face substantially elevated risks of respiratory distress syndrome, sepsis, neurological impairment, and long-term developmental delays (Mead et al., 2023). Therefore, identifying the causes of PTB, including its molecular underpinnings, remains an important public health goal.

Preterm birth (PTB) is a complex condition that is commonly stratified by either clinical presentation and/or gestational age at delivery. Three primary clinical phenotypes have been identified to be presented clinically and include; medically indicated PTB (inPTB), idiopathic PTB (iPTB), and preterm pre-labor rupture of membranes (PPROM). Idiopathic PTB and PPROM share a spontaneous onset of labor without medical induction and are therefore commonly classified together as spontaneous PTB (sPTB) (Mead et al., 2023). The World Health Organization also stratifies PTB by gestational age: extreme PTB (<28 weeks gestation), very preterm (28 to <32 weeks’ gestation), and moderate or late PTB (32 to <37 weeks gestation) (World Health Organization, 2023).

These classifications indicate that PTB is a heterogeneous syndrome in which multiple biological pathways prematurely activate parturition, rather than a single condition (Romero et al., 2014). Key biological mechanisms include infection-driven inflammation, placental vascular disease, decidual dysfunction, immune intolerance, and altered progesterone signaling, which may act independently or in combination to trigger labor or membrane rupture (Romero et al., 2014). Structural abnormalities of the reproductive tract, such as cervical insufficiency, uterine malformations, or intrauterine scarring, may further predispose to early cervical dilation or membrane rupture (Nold et al., 2012). Collectively, these pathways are shaped by complex interactions among genetic, environmental, behavioral, and social determinants, with prior spontaneous PTB remaining the strongest predictor of recurrence (Van Gils et al., 2024).

Rapid advances in genomics, transcriptomics, and gene-editing technologies have enabled the precise identification of genes, regulatory loci, and expression signatures that contribute to the pathophysiology of preterm birth (Mead et al., 2023). However, identifying potential loci for intervention of preterm birth requires careful consideration of both function and evolutionary constraint because genes involved in gestation length, placental function, and parturition are subject to strong selective pressures (Mika et al., 2021). Consequently, not all genomic regions are equally amenable to modification. Disrupting highly conserved or pleiotropic genes could have unintended consequences for fetal development or maternal health. Thus, a major challenge in applying gene-editing or genomic modulation strategies lies in distinguishing causal loci that can be safely and effectively targeted to reduce preterm birth risk.

To address this challenge, evolutionary biology provides a valuable guiding framework. Human gestation and birth are the products of millions of years of reproductive adaptation shaped by trade-offs among fetal growth, maternal pelvic anatomy, metabolic demands, and ecological pressures (Stansfield et al., 2021). The obstetrical dilemma, attributes the timing of human birth to a fundamental biomechanical conflict between the narrow pelvis required for efficient bipedal locomotion and the wide pelvis needed to deliver a large-brained infant, a trade-off that constrains the degree of neurodevelopmental maturity at which human neonates are born relative to other primates (Furness, 2025; Washburn, 1960). A complementary but distinct framework, the Energetics of Gestation and Growth hypothesis (Dunsworth et al., 2012), proposes that birth timing is driven instead by the limits of maternal metabolic capacity to sustain continued fetal growth, balancing the mother’s physiological constraints against the infant’s developmental needs.

Similarly, evolutionary forces that once enhanced reproductive fitness can now contribute to disease susceptibility. For instance, genetic variants that historically conferred stronger immune defenses in the reproductive tract may now predispose certain individuals to inflammation-driven preterm birth (LaBella et al., 2020). Case–control studies in African-American women and infants have identified immune-related polymorphisms, particularly in genes that encode cytokines such as *IL-15* and *IL2RB*, as significant predictors of spontaneous preterm birth, suggesting that alleles once advantageous for infection resistance may now contribute to higher PTB rates through heightened inflammatory responses (Velez et al., 2009). Moreover, population-level evolutionary analyses reveal that the progesterone receptor *(PGR)* gene has undergone recent positive selection, with East Asian populations nearly fixing a derived allele associated with altered gene expression and increased risk of early spontaneous preterm birth, whereas European populations maintain greater PGR diversity under balancing selection (Li et al., 2018). Examining these processes through an evolutionary lens would provide better discernment of which genomic regions are under strong selective constraint and thus risky to perturb and which may be more flexible targets for intervention. Further integrating evolutionary insights with machine learning will offer a robust way prioritizing functional loci and identifying regulatory pathways that can be targeted to prevent preterm birth.

### Evolution of genes involved in preterm birth

**FIGURE 1:**
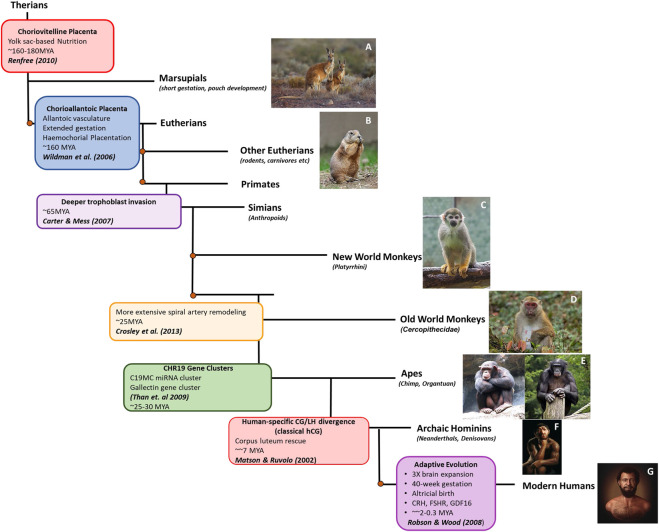
Major Evolutionary and genomic events in the evolution of human pregnancy and parturition. This figure shows the key events (in a phylogenetic framework) in the evolution of human pregnancy, including the emergence of the hemochorial placenta, an increase in body and brain size, the emergence of HCG, CHR19 MC cluster, adaptive evolution of key genes, etc. (Maston and Ruvolo, 2002; Wildman et al., 2006; Than et al., 2009; Crosley et al., 2013; Carter and Mess, 2007; Robson and Wood, 2008; Renfree, 2010) [**(A)** = One adult and one young Red Kangaroo; **(B)** = Black-tailed prairie dog (*Cynomys ludovicianus);* **(C)** = Common squirrel monkey (*Saimiri sciureus*); **(D)** = Assam macaque *(Macaca assamensis)*, **(E)** = Chimpanzee (left) and Bonobo (right). **(F)** = *Homo neanderthalensis*, **(G)** = Modern Human face]. All images are licensed under *CC BY 4.0* and *CC BY 3.0* (Delso, 2012; Moraes et al., 2018; Mukherjee, 2024; PotMart186 and One adult and one young Red Kangaroo at Sturt National Park NSW, 2021; Ravi, 2011; Rennett, 2019).

From an initial list of approximately 1,500 genes implicated in pregnancy and parturition (Supplementary Table S1), and against the backdrop of key evolutionary events depicted in Figure 1, we identified a subset exhibiting evidence of lineage-specific evolutionary change, including signatures of positive selection, gene duplication, and transposable element-mediated innovation. Critically, inclusion in Table 1 was based primarily on evolutionary evidence rather than genetic association with preterm birth, many of the evolutionary studies cited do not explicitly link these loci to preterm birth outcomes. Rather, their established roles in hormonal signalling, immune regulation, placentation, and uterine activation place them within the biological pathways that underlie susceptibility to spontaneous preterm birth. Where available, human genetic association data, including odds ratios and p-values from candidate gene and genome-wide association studies, are presented as corroborating evidence that evolutionary variation at these loci translates into measurable phenotypic consequences relevant to PTB risk. However, for several loci the association evidence remains preliminary, population-specific, or limited to a single cohort, and many findings require replication across independent and ancestrally diverse populations before firm conclusions can be drawn.

**TABLE 1:** Genes and genomic loci with lineage-specific evolutionary change and evidence of association with preterm birth.

Code	Gene	Chr	rsID	PTB phenotype/Relevance	Effect size (OR, 95% CI)	P-value	Evolutionary/Functional evidence
Hormonal Signalling							
HGA	*PGR*	11	rs660149	sPTB <37 weeks	OR = 2.3 (95% CI 1.2–4.5) (Langmia et al., 2015c)	0.011	Positive selection in N-terminal IF domain; human-specific substitutions enabling functional progesterone withdrawal (Chen et al., 2008)
HGA	*PGR*	11	rs471767	sPTB; iPTB	OR = 1.85 (95% CI 1.04–3.26) (Manuck et al., 2010)	0.035	Positive selection in N-terminal IF domain (Chen et al., 2008)
FEV	*ESR1*	6	NA	Reproductive endocrinology	NA	NA	Strong purifying selection; essential role in reproductive physiology (Weckle et al., 2017)
FEV	*ESR2*	14	NA	Estrogen receptor signalling	NA	NA	Episodic positive selection in ligand-binding domain; enhanced ERβ activity (Weckle et al., 2017)
LGA	*CRHBP*	5	rs7756445	RH pathway regulation; associated with gestational age via DNA methylation; identified in multiple PTB databases (Li et al., 2023)	NA	NA	Anthropoid-specific THE1B retroviral LTR drives placental CRH expression (Dunn-Fletcher et al., 2018)
HGA	*FSHR*	2	rs6166	sPTB	OR = 2.52 (95% CI 1.20–5.33) (Dominguez-Lopez et al., 2018)	0.02	Accelerated evolution on human lineage; altered FSH sensitivity (Plunkett et al., 2011)
HGA	*FSHR*	2	rs9789744	sPTB	OR = 0.56 (95% CI 0.40–0.78) (Plunkett et al., 2011)	6.78 × 10^−4^	Accelerated evolution on human lineage; altered FSH sensitivity (Plunkett et al., 2011)
HGA	*FSHR*	2	rs11686474	sPTB	OR = 1.82 (95% CI 1.32–2.52) (Plunkett et al., 2011)	2.72 × 10^−4^	Accelerated evolution on human lineage (Plunkett et al., 2011)
HGA	*RLN2*	9	rs4742076	PPROM; sPTB	OR = 11.0 (95% CI 2.0–60.5) (Rocha et al., 2013)	0.001	Gene duplication in catarrhine primates; convergent evolution (Arroyo et al., 2014)
HGA	*RLN2*	9	rs3758239	PPROM; sPTB	OR = 12.5 (95% CI 2.7–50.0) [PPROM]; OR = 7.14 (95% CI 1.7–25.0) [sPTB] (Rocha et al., 2013)	0.0002/0.003	Gene duplication in catarrhine primates (Arroyo et al., 2014)
Immune Regulation							
LGA	*TLR4*	9	rs4986790	PTB (<35 weeks)	NA	0.036 (Lorenz et al., 2002)	Lineage-specific positive selection; host–pathogen arms race (Quach et al., 2013)
LGA	*TLR4*	9	rs4986791	PTB (<33 weeks)	NA	0.044 (Lorenz et al., 2002)	Host–pathogen coevolution 10]
HGA	*IL15*	4	rs10833	iPTB	OR = 0.30 (95% CI 0.14–0.62) (Velez et al., 2009)	0.001	Population-specific positive selection in African ancestry (Fumagalli et al., 2009)
HGA	*IL2RB*	22	rs84460	iPTB	OR = 2.32 (95% CI 1.47–3.67) (Velez et al., 2009)	<0.001	Rapid evolution in hominids; African-specific selection (Fumagalli et al., 2009)
HGA	*IL6*	7	rs1800795	iPTB; sPTB	OR = 2.8 (95% CI = 1.2–6.4) (Pereyra et al., 2016)	<0.05	Balancing selection maintaining allelic diversity (Furness, 2025)
HGA	*IL10*	1	rs1800896	iPTB; PPROM	OR = 2.1 (95% CI NR) (Annells et al., 2004)	0.04	Balancing selection; regulatory variation (Furness, 2025)
LGA	*TNF*	6	rs1800629	sPTB; iPTB	OR = 1.74 (95%CI = 1.04-2.90) (Harper et al., 2011)	0.03	Balancing selection; rapid evolution (Furness, 2025)
HGA	*HLA-DQA1*	6	rs9272346	sPTB	OR = 0.65 (95% CI 0.46–0.94) (Falah et al., 2013)	0.022	Balancing selection maintaining MHC diversity (Falah et al., 2013)
FEV	*KIR genes*	19	NA	maternal–fetal immune tolerance	NA	NA	Rapid species-specific expansion; positive selection (Abi-Rached et al., 2010)
Placentation							
FEV	*C19MC*	19	NA	Placental development	NA	NA	Primate-specific Alu-mediated duplications; paternal imprinting (Malnou et al., 2018)
FEV	*PSG genes*	19	NA	Maternal–fetal tolerance	NA	NA	Primate-specific expansion; positive selection in N-terminal domains (Yang et al., 2021)
FEV	*CGB genes*	19	NA	Pregnancy maintenance	NA	NA	Duplication from LHB in anthropoid primates (Maston and Ruvolo, 2002)
FEV	*LGALS13*	19	NA	Maternal–fetal tolerance	NA	NA	Primate-specific TE-mediated duplication; positive selection in CRDs (Than et al., 2009)
Membrane Integrity and Parturition							
LGA	COL1A1	17	rs1061237	iPTB; PPROM	NA	0.04 (Velez et al., 2008)	Positive selection: dN/dS 0.4215 vs. 0.1656 (p < 0.001) (Stempfle et al., 2013)
LGA	COL5A1	9	rs12005720	PTB	NA	0.04 (Velez et al., 2008)	Differential selection: dN/dS 0.0611 vs. 0.0368 (p = 0.003) (Stempfle et al., 2013)
HGA	ADAMTS2	5	rs59567206	Membrane rupture/PPROM	NA	0.000141 (Stempfle et al., 2013)	Differential selection: dN/dS 0.0741 vs. 0.0505 (p = 0.0006) (Stempfle et al., 2013)
HGA	MMP1	11	rs1939008	iPTB	NA	0.03 (Velez et al., 2008)	Lineage-specific changes; primate divergence (Pan et al., 2022; Fanjul-Fernández et al., 2010)
LGA	MMP3	11	rs520540	iPTB	NA	0.01 (Velez et al., 2008)	Positive selection; primate divergence (Pan et al., 2022; Fanjul-Fernández et al., 2010)
HGA	MMP9	20	rs3918242	PTB (<37 weeks)	OR = 2.61 (95% CI 1.46–4.69) (Pandey and Awasthi, 2019)	0.001	Positive selection (12 sites); rapid primate evolution (Pan et al., 2022; Fanjul-Fernández et al., 2010)
HGA	PTGS1	9	rs10306227	PTB	OR = 0.60 (95% CI 0.38–0.93) (Liu et al., 2012)	0.02	Independent COX duplications in vertebrates (Pan et al., 2022; Fanjul-Fernández et al., 2010)
HGA	PTGS2	1	rs5276	PTB; iPTB	OR = 0.40 (95% CI 0.18–0.86) (Liu et al., 2012)	0.02	Independent COX duplications; inducible expression (Järving et al., 2004)
HGA	PTGER3	1	NA	NA	NA	0.02	Adaptive evolution in prostaglandin signaling (Järving et al., 2004)

Column Definitions.

Gene Location: Gene boundaries in base pairs (GRCh38/hg38); Evolutionary Evidence: Description of evolutionary signature; Association Study Citation: Study reporting genetic association with PTB; Evolutionary Citation: Study(ies) documenting evolutionary selection or comparative genomics evidence.

Evidence codes: HGA, human genetic association evidence; LGA, Limited or inconsistent genetic association evidence (single unreplicated study, p-value only, directionally inconsistent across populations, or continuous outcome only); FEV, Functional/evolutionary evidence only.

Abbreviations: Chr, chromosome; dN/dS, ratio of non-synonymous to synonymous substitutions; GA, gestational age; IF, inhibitory function; iPTB, indiopathic preterm birth; LTR, long terminal repeat; PE, preeclampsia; PPROM, preterm premature rupture of membranes; PTB, preterm birth; sPTB, spontaneous preterm birth; OR, odds ratio from identified studies; CI, confidence interval; NA, not applicable.

### Progesterone receptor gene (*PGR*)

Progesterone plays a central role in maintaining uterine quiescence throughout pregnancy, primarily through the action of the progesterone receptor (*PGR*), a nuclear transcription factor that regulates genes involved in inflammation, myometrial contractility, and cervical remodeling (Tripathy et al., 2022). The disruption or premature attenuation of progesterone signaling is suggested to be a key mechanism of spontaneous preterm birth (Pisacreta and Mannella, 2022). Comparative evolutionary analysis revealed that PGR has undergone adaptive evolution in primates, with the recently evolved human and chimpanzee lineages showing a significant excess of non-synonymous substitutions relative to synonymous changes–a hallmark of positive selection (Chen et al., 2008). This pattern was, however, weak or absent in other mammals, pointing out the unique selective pressures on primate *PGR*. Notably, many of these human-specific amino acid substitutions occur in the N-terminal inhibitory function (IF) domain of *PGR*, a region that modulates transcriptional activity and cofactor interactions (Chen et al., 2008). These substitutions are nearly fixed in humans and are thought to facilitate functional progesterone withdrawal, a primate-specific mechanism in which progesterone levels remain high at term while downstream signaling through *PGR* is attenuated, permitting the onset of labor (Ilicic et al., 2019).

Building on evolutionary evidence implicating PGR in the regulation of parturition, genetic association studies consistently link common PGR variation to spontaneous preterm birth. Early candidate gene analyses examined both maternal and fetal PGR variation across multiple SNPs, identifying significant associations at several loci for both mothers and their preterm infants, suggesting a locus-wide signal across the PGR gene rather than a single causal variant (Ehn et al., 2007). Subsequent studies reiterated and extended these findings, demonstrating that women with a personal or family history of preterm birth were significantly more likely to carry minor alleles at key PGR SNPs, with associations particularly pronounced for earlier gestational age at delivery (Manuck et al., 2010). Independent replication across ethnically distinct cohorts further corroborated these findings, with fetal PGR variation, notably rs1942836, showing consistent association with spontaneous preterm birth across populations, supporting the existence of both maternal and infant PGR effects on gestational timing (Mann et al., 2013).

### Estrogen receptors (*ESR1* and *ESR2*)

Estrogen signaling is essential during the initiation of labor by promoting uterine contractility and cervical ripening as pregnancy progresses (Bakker et al., 2017). Evolutionary analyses of the estrogen receptor genes reveal marked divergence between *ESR1* (ERα) and *ESR2* (ERβ) in primates. *ESR1* is highly conserved under strong purifying selection across primate lineages, reflecting its essential and non-redundant role in reproductive physiology (Weckle et al., 2017). In contrast, *ESR2* exhibits lineage-specific adaptive evolution, with episodic positive selection observed in the New World monkey lineage. Functional ancestral gene resurrection and comparative assays demonstrate that these adaptive changes, particularly within the ligand-binding domain, enhanced ERβ transcriptional activity in response to estradiol, likely as an adaptation to the unusually high circulating steroid levels characteristic of New World monkeys (Weckle et al., 2017).

### Toll-like receptors

Toll-like receptors (TLRs) are central components of innate immunity that recognize pathogen-associated molecular patterns and activate inflammatory signaling (Kircheis and Planz, 2023). Consistent with long-standing host–pathogen arms races, TLR genes have been frequent targets of positive selection in primate evolution. Comparative analyses show that although many TLRs are constrained by purifying selection due to their essential roles in host defense, specific codon sites have repeatedly undergone lineage-specific positive selection, indicating adaptive fine-tuning of receptor function in response to distinct microbial ecologies (Quach et al., 2013). A particularly illustrative contrast is *TLR5*, the receptor for bacterial flagellin. In chimpanzees, *TLR5* is under strong purifying selection, highlighting its conserved importance for survival, whereas in humans, a common loss-of-function stop-codon variant, dominant-negative, has risen to high frequency and appears to have been favored by selection in certain populations, potentially reflecting an alternative strategy that dampens flagellin-driven inflammation (Quach et al., 2013).

These interspecies differences are directly relevant to pregnancy, where excessive or mistimed TLR activation at the maternal–fetal interface can amplify inflammatory cascades and promote preterm uterine activation. In particular, *TLR4*-mediated responses to bacterial endotoxin are a well-established pathway implicated in infection-induced preterm labor, suggesting that primate- and human-specific evolutionary trajectories in innate immune genes may shape susceptibility to pregnancy-related infections (Liu et al., 2007; Elovitz et al., 2003). Consistent with this pathway, multiple studies report associations between maternal and fetal TLR variants and spontaneous preterm birth phenotypes, including spontaneous PTB, infection-associated PTB, and preterm premature rupture of membrane (Langmia et al., 2015a; Langmia et al., 2015b; Salem et al., 2016; Ramos et al., 2016).

### Chromosome 19 microRNA cluster

The Chromosome 19 microRNA cluster (C19MC) represents the largest microRNA gene cluster in the human genome, spanning approximately 100 kb on chromosome 19q13.4 and comprising 46 microRNA genes with nearly exclusive placental expression (Malnou et al., 2018). This remarkable genomic feature emerged within the primate lineage and is present in anthropoid primates, including both New World and Old World monkeys as well as apes, while being conspicuously absent in strepsirrhine primates and other mammals, marking it as a relatively recent evolutionary innovation (Malnou et al., 2018). The expansion of the cluster is found to have been driven by transposable elements, as evidenced by the dense interspersion with ancient *Alu* repeats, which likely facilitated the tandem duplication events responsible for cluster growth during an early *Alu* expansion wave (Lehnert et al., 2009).

Functionally, C19MC microRNAs are highly abundant in trophoblastic cells of the human placenta and are subsequently released into maternal circulation via exosomes (Malnou et al., 2018). These microRNAs exhibit paternal expression and maternal imprinting, revealing an evolutionary tug-of-war consistent with genomic imprinting theory and paternal interests in maximizing fetal growth (Noguer-Dance et al., 2010). It has further been demonstrated that C19MC miRNAs enable the placenta to fine-tune maternal immune responses. Specifically, products of the C19MC cluster suppress excessive innate immunity in trophoblast tissue (Mouillet et al., 2025). When C19MC expression is experimentally silenced in placental cells, these cells exhibited exaggerated responses to viral RNA mimics through TLR3, an antiviral Toll-like receptor. Thus, C19MC miRNAs appear to dampen TLR3-mediated inflammatory signaling in the placenta, preventing excessive immune reactions to viral elements (Xie et al., 2014). This regulatory mechanism is adaptively significant, as unchecked antiviral responses in the placenta could compromise fetal development or trigger preterm labor. Additionally, C19MC miRNAs have been implicated in controlling trophoblast invasion and migration, as well as mediating adaptation to hypoxic stress in the placenta (Mong et al., 2020).

The evolution of C19MC thus represents an innovative primate-specific strategy to protect pregnancy through enhanced viral resistance and immunomodulation at the maternal-fetal interface. These miRNAs may reduce the risk of infection-driven pregnancy complications, including preterm birth, while simultaneously safeguarding fetal development (Dumont et al., 2017). Population-level differences in C19MC expression or sequence variation could potentially influence resilience to specific viral or inflammatory triggers of preterm birth, establishing this cluster as an important subject of ongoing research in evolutionary medicine.

### Chromosome 19 genes

Beyond C19MC, human chromosome 19 harbors several gene families that have expanded or diverged in primates and are implicated in maintaining pregnancy, promoting fetal growth, and facilitating immune tolerance, all of which are factors contributing to the risk of preterm birth. A prominent example is the pregnancy-specific glycoprotein (PSG) family (Mann et al., 2022). PSGs are immunomodulatory proteins secreted by the placental syncytiotrophoblast and contribute to maternal-fetal immune tolerance and vascular remodeling in pregnancy. In humans, the PSG genes form a tandem cluster of 11 genes on chromosome 19q13.2 (Yang et al., 2021). Comparative genomics indicates that this is a result of primate-specific gene expansions. Rodents also evolved their own PSG-like genes, but notably, the expansion of PSG gene families occurred independently in primates and rodents, a case of convergent evolution to meet the demands of placental reproduction (McLellan et al., 2005). The rapid diversification of PSGs in primates suggests positive selection, possibly driven by ever-changing pathogens or maternal immune pressures, acting on their N-terminal domains, which interact with immune cells (McLellan et al., 2005). This means that different primate species have distinct repertoires of PSG variants, potentially specialized to their particular reproductive environments. The functional consequence is that PSGs have continuously evolved to better modulate the maternal immune system, and any dysregulation in this delicate immune balance can predispose to conditions like preterm labor.

Humans and other anthropoid primates produce chorionic gonadotropin (hCG), a hormone crucial for maintaining early pregnancy, which has been considered an essentially enhanced form of luteinizing hormone (LH) adapted to act over a longer duration (Mann et al., 2022). Genetically, hCG’s β-subunit is encoded by a cluster of genes that arose from duplication of the LHβ gene. The *LHB/CGB* gene cluster on 19q13.3 contains one luteinizing hormone β (*LHB*) gene and six chorionic gonadotropin β (*CGB*) genes in humans (Mann et al., 2022; Maston and Ruvolo, 2002). These duplications arose during primate evolution and are absent in most non-primates, enabling higher and more sustained CG production that extends luteal support of pregnancy beyond what LH alone could achieve. This was an adaptive response as anthropoid primates maintain hemochorial placenta that demands prolonged progesterone support from the corpus luteum in early gestation (Wildman et al., 2006; Elliot and Crespi, 2009). The evolution of multiple CGB gene copies, with slight variations in expression and regulation, likely provided a selective advantage by securing the maternal recognition of pregnancy and reducing early pregnancy loss (Henke and Gromoll, 2008). However, variation in these genes or their regulatory regions could alter hCG production and compromise luteal support, potentially increasing susceptibility to preterm labor or miscarriage.

Chromosome 19 is also renowned for housing the killer-cell immunoglobulin-like receptor (KIR) gene complex. KIR genes encode receptors on uterine natural killer cells that engage with fetal HLA-C molecules on placental cells, regulating trophoblast invasion and arterial remodeling. The KIR region has undergone rapid, species-specific expansion and diversification in primates (Kelley et al., 2005). Notably, chromosome 19 also harbors a primate-specific cluster of galectin genes that emerged through duplication and rearrangement mediated by transposable elements during anthropoid evolution (Than et al., 2009). These galectins, which recognize cell-surface glycans to regulate immune responses, show a striking parallel to KIR evolution: both gene families exhibit lineage-specific expansion, evidence of positive selection, and functional diversification in carbohydrate- or ligand-recognition domains. Three human galectins in this cluster (*LGALS13*, -*14*, and -*16*) are placenta-specific and predominantly expressed by the syncytiotrophoblast, where they induce apoptosis of maternal T lymphocytes, potentially providing an additional layer of immune tolerance at the maternal-fetal interface (Than et al., 2009). Only a subset of KIR genes are conserved between humans and chimpanzees; others are lineage-specific, reflecting rapid evolution shaped by the competing demands of immune defense and maternal-fetal tolerance (Kelley et al., 2005). This co-evolution of KIR and HLA genes directly influences pregnancy outcomes. Certain KIR/HLA combinations are linked to preeclampsia and could similarly affect preterm birth risk through disrupted placental development. The primate-specific expansion of KIRs highlights an evolutionary balancing act between immune surveillance and placental invasion. When this balance fails, whether the immune response is overly aggressive or insufficiently supportive, pregnancy complications can result.

## Gestational variation across vertebrates

### Mammalian gestation diversity

Mammals nourish embryos internally through the placenta and uterus. Among mammals, eutherians (i.e., placental mammals) have the longest intrauterine development, while marsupials follow an alternative strategy that is, brief pregnancies followed by prolonged pouch-based postnatal development (Funston et al., 2022). Gestation length in placental mammals generally increases with body size and neonate maturity. A tiny golden hamster gestates for about 2 weeks, while a 3,000-kg blue whale carries its fetus for approximately 11 months and an African elephant for 21–22 months. Primates have disproportionately longer gestation periods relative to their body size, typically producing single, well-developed infants via hemochorial placentation. Small, strepsirrhine lemurs gestate for under 3 months, whereas Old World monkeys and apes range from 5 to 9 months (Funston et al., 2022). An exception to this pattern is the tarsier, a small bodied <200 g prosimian primate that exhibits a 6 months gestation pattern (Jameson et al., 2011). Rodents exhibit the shortest gestation periods among placental mammals. House mice gestate for only 19–21 days before delivering 5–8 altricial pups. Their labyrinthine hemochorial placenta enables rapid fetal development, with gestations up to 50% shorter than species with less-interdigitated placental structures (Panja et al., 2021). However, guinea pigs weighing about 1 kg, represent an exception, gestating for 65–70 days and producing precocial offspring (Funston et al., 2022).

Carnivores show intermediate gestation lengths (2–2.5 months for cats and dogs) supported by a zonary endotheliochorial placenta, with exception of the hemochorial placentas exhibited by hyenas (Funk et al., 2019). Many carnivore species employ embryonic diapause: mustelids and bears mate in spring or summer, but embryos remain dormant for months before implanting, ensuring offspring are born in favorable seasons despite relatively short post-implantation gestation (about 60 days) (Panja et al., 2021). Distantly related mammal species typically referred to as ungulates (i.e., horses, cows, sheep, pigs, elephants, etc.) often have long gestations that produce precocial young capable of standing or running shortly after birth. Sheep gestate for approximately 150 days, cattle for 280 days, horses for 11 months, and elephants for 22 months. The epitheliochorial (non-invasive) placenta seen in artiodactyls and perissodactyls may necessitate longer gestation to achieve fetal maturity equivalent to species with more invasive placental types (Das et al., 2023).

Furthermore, whales and dolphins (i.e., cetaceans) exhibit prolonged gestations ranging from 10 to 18 months, with killer whales gestating for 16–18 months. The aquatic environment demands immediate swimming competence at birth, requiring extended intrauterine development. Their diffuse epitheliochorial placenta supports this strategy, producing large, neurologically mature calves (Funston et al., 2022). In stark contrast, marsupials employ an abbreviated gestation strategy, birthing highly altricial young after only 8–40 days of intrauterine development. Virginia opossums gestate for merely 12–13 days before delivering neonates weighing about 0.2 g that complete development ex utero by crawling to the maternal pouch. Red kangaroos gestate for ∼33 days, producing jellybean-sized joeys that undergo 6–8 months of pouch-dependent maturation. This strategy reflects the limitations of their rudimentary yolk-sac placenta, which cannot support extended intrauterine growth (Funston et al., 2022).

### Other vertebrate gestation diversity

Birds are exclusively oviparous, with incubation periods ranging from 10 to 14 days in small songbirds to 80–85 days in wandering albatrosses and brown kiwis. Incubation duration correlates with developmental state as altricial chicks (songbirds, hawks) hatch quickly but undeveloped, whereas precocial chicks (ducklings, game birds) spend longer in eggs and hatch with downy feathers and mobility. No birds evolved viviparity, likely due to flight constraints (Izquierdo et al., 2025). Reptiles, on the other hand, show diverse reproductive modes. Most are oviparous, with sea turtles having about 50–70 days of incubation, but have their viviparity evolved in over 100 squamate lineages. The cold-climate hypothesis explains this as gravid females can thermoregulate by basking, keeping embryos warmer than nest temperatures, shortening development time. (Warren et al., 2021). European adders in Scandinavia are viviparous with about a 4-month gestation. Pythons provide remarkable parental care, coiling around eggs and generating metabolic heat through muscle contractions, maintaining eggs above ambient temperature for approximately2 months (Warren et al., 2021).

Amphibians further display extraordinary reproductive diversity (Vági and Székely, 2023). Most anurans are oviparous with brief embryonic development; bullfrog eggs hatch in approximately 4–5 days, yielding free-swimming tadpoles. In stark contrast, the alpine salamander (*Salamandra atra*) exhibits the longest gestation period among tetrapods (2–4 years at high elevations), producing two fully metamorphosed juveniles that bypass larval aquatic stages. Alternative reproductive modes include dorsal brood pouches in marsupial frogs (several weeks’ development), dermal incubation chambers in Surinam toads (about 3 months), and gastric brooding in the extinct *Rheobatrachus* species, which suppressed acid secretion while brooding tadpoles for about 6–7 weeks. Teleost and elasmobranch fishes display extensive reproductive variation. While oviparity with external fertilization predominates, viviparity has arisen independently in over 20 fish lineages. Many sharks exhibit yolk-sac viviparity with gestation periods of 9–12 months; the frilled shark (*Chlamydoselachus anguineus*) has among the longest vertebrate gestations at approximately 3.5 years. Poeciliid fishes such as guppies employ lecithotrophic viviparity with about 22–30 days gestation. Male pregnancy in syngnathids (seahorses and pipefish) represents a unique adaptation: males incubate eggs in specialized brood pouches for 10 days to 6 weeks, providing respiratory gas exchange, osmoregulation, and nutritional provisioning via placenta-analogous tissue prior to releasing fully formed juveniles (Vági and Székely, 2023). Finally, onychophoran velvet worms are among the odd assemblage of invertebrates that exemplify live birth and the appearance of placenta-like structures (Blaxter and Sunnucks, 2011).

### Evolutionary pressures shaping gestation

#### Body size, metabolism, and energetic ceilings

Body size is one major predictor of gestation length across many taxa. Larger mothers generally (e.g., New World monkeys, Old World monkeys, Rodentia, Carnivora, Cetacea + Artiodactyla (i.e., Cetartiodactyla)) require longer gestations or incubation periods to produce viable offspring (Danis and Rokas, 2024). Evolutionary modeling has detected 52 significant shifts in gestation length across eutherians, with direction and frequency varying substantially by order (Danis and Rokas, 2024). For example, bats showed primarily negative shifts, consistent with metabolic constraints of flight, while marine mammals (pinnipeds and cetaceans) independently evolved toward longer gestation, suggesting convergent selection in aquatic environments (Danis and Rokas, 2024). However, in endotherms, elevated and stable body temperatures can accelerate embryonic development relative to ectotherms, though egg/offspring size still imposes time requirements for organogenesis and growth (Tzschen et al., 2011).

In humans, the timing of birth appears to be constrained by maternal metabolic capacity as well as pelvic dimensions alone. The energetics of gestation and growth (EGG) hypothesis proposes that human gestation terminates when maternal energy expenditure approaches a physiological ceiling, preventing further intrauterine fetal growth (Dunsworth et al., 2012). This framework recontextualizes the traditional obstetrical dilemma, which posits an evolutionary trade-off between pelvic dimensions for bipedalism and neonatal head size. While pelvic constraints undoubtedly influence parturition, contemporary evidence suggests they do not fully account for either human gestational timing or the relatively immature state of human neonates at birth (Dunsworth et al., 2012).

### Developmental state at birth

Gestation length depends heavily on how developed offspring need to be at birth. Species with precocial young, born mature, mobile, and capable, require longer pregnancies and typically have smaller litters. Species with altricial young, born underdeveloped and dependent, can have shorter pregnancies and often larger litters, though these babies need extensive care after birth (Kraus et al., 2005). Also, environmental pressures shape which strategy works best. Species living in open habitats where predators are a constant threat benefit from having precocial babies that can run or move quickly right after birth (Taylor et al., 2022). In contrast, species that can hide their young in burrows, nests, or dens can afford to have altricial babies because parents can protect and feed them while they develop (Kappeler and Kappeler, 2021).

### Placental (and placenta-like) exchange (nutrient transfer and gestation potential)

The physical connection between mother and developing embryo determines how long pregnancy can last and how developed offspring will be at birth. Placentas in mammals are classified by how closely maternal and fetal blood come into contact. In epitheliochorial placentas, multiple tissue layers keep the two blood supplies separate. Endotheliochorial placentas have fewer barriers. Hemochorial placentas, the most invasive type, allow fetal tissues to sit directly in maternal blood, enabling highly efficient exchange of nutrients and waste (Haeusler et al., 2021). Mammals are not the only animals to evolve placenta-like structures. Live birth and specialized ways of feeding developing embryos have evolved repeatedly across reptiles, fish, and amphibians. These nutritional strategies include placentotrophy (placenta-like nutrient transfer), histotrophy (absorbing secretions from the mother’s reproductive tract), oophagy (embryos eating unfertilized eggs), and embryophagy (embryos eating other embryos) (Blackburn, 2015). One of the most remarkable examples of convergent evolution is male pregnancy in seahorses and pipefish. Males do not just carry the eggs—their brood pouches actively support development by exchanging oxygen and waste gases, regulating salt and water balance, and even providing some nutrients to the growing embryos (Whittington et al., 2015).

### Environmental seasonality, delayed implantation, and embryonic diapause

In environments with distinct seasons, timing birth to coincide with abundant food provides major survival advantages. One strategy is delayed implantation, where animals mate at one time but the embryo does not attach to the uterus and begin developing until months later. This decouples mating from birth timing. Early research found delayed implantation in 47 mammal species across 10 families, evolving independently at least 17 times (Sandell, 1990). More recent studies show this phenomenon is even more widespread and emphasize that embryonic diapause is a common strategy for dealing with unpredictable food and energy availability (Whittington et al., 2015; Sandell, 1990). Similar timing strategies appear in fish and amphibians, showing that seasonal environments consistently favor mechanisms that protect reproduction from environmental unpredictability (Renfree and Fenelon, 2017; Fenelon and Renfree, 2018).

### Physiological and anatomical constraints

Some reproductive strategies are limited by intrinsic constraints rather than ecology alone. In humans, competing frameworks, pelvic constraints (obstetrical dilemma) versus maternal metabolic ceilings (EGG), both highlight that gestation cannot extend indefinitely without maternal costs, and that neonate immaturity can emerge from constraint-driven trade-offs (Dunsworth et al., 2012; Haeusler et al., 2021). In reptiles, developmental mechanisms can constrain transitions to viviparity. For example, theory and comparative evidence connect viviparity with shifts in sex-determination systems: because temperature-dependent sex determination (TSD) relies on environmental incubation temperatures, internal gestation can select for genotypic sex determination (GSD) to stabilize sex ratios under maternal thermoregulation (Cornejo-Páramo et al., 2020).

### Detecting adaptive evolution in preterm birth (PTB)

Studying pregnancy from an evolutionary perspective has revealed that certain pregnancy-related genes show signs of natural selection. Researchers use two main approaches: comparing genes across different species and examining genetic variation within human populations. For example, studies comparing gene activity in placentas across species identified 115 genes that are active in all placental mammals (Armstrong et al., 2017). These core genes include factors that regulate immune responses (like annexins and S100 proteins), proteins involved in cell attachment and invasion, and factors that help form the syncytium, the fused cell layer in the placenta (Armstrong et al., 2017). The presence of such immune and invasion genes in every placenta studied points out the strong purifying selection that maintains essential pregnancy functions like maternal-fetal immune tolerance and trophoblast invasion. At the same time, evolutionary divergence is evident. It reported that multiple preeclampsia-associated genes are differentially expressed in the human placenta compared to other mammals. This suggests human-specific regulation of placental pathways, potentially an adaptation to our species’ unique reproductive challenges. Additionally, the expression of some genes correlates with placental morphological differences across species, further highlighting how divergent selection has tailored placental biology to different evolutionary lineages (Armstrong et al., 2017).

There have also been identified signatures of positive selection in genes tied to birth timing by comparing DNA sequence evolution across primates. A recent study hypothesized that the unusually large neonatal head and bipedal-narrowed pelvis of humans created evolutionary pressure for shorter gestation to avoid obstructed labor (Plunkett et al., 2011). Indeed, human gestation length appears shortened relative to other primates (based on allometric scaling) and many reproduction-related genes show accelerated sequence evolution on the human lineage (Plunkett et al., 2011). In other words, these parturition genes accumulated amino acid changes at a higher rate than expected, consistent with positive selection for altered function (Plunkett et al., 2011). Notably, several of these accelerated genes are involved in pregnancy maintenance, fetal development, or hormonal signaling (Plunkett et al., 2011). It was also suggested that polymorphisms in the follicle stimulating hormone receptor (*FSHR*) were enriched in mothers with spontaneous PTB with the top associations in *FSHR*. *FSHR* had not previously been linked to parturition, so its identification underscores how adaptive evolution analysis can reveal new candidates for PTB (Plunkett et al., 2011). The implication is that some genes essential for human birth timing underwent functional changes during human evolution, and modern genetic variation in those genes influences PTB susceptibility.

Evolutionary conflict and cooperation between the mother, fetus, and pathogens have also left their mark on pregnancy genes. In particular, genes at the maternal-fetal interface often show signs of rapid evolution, driven by the need to balance maternal immune defense with tolerance of the semi-allogeneic fetus. The placenta’s role as an immunological barrier has led to adaptations in immune regulatory genes; for instance, HLA and killer immunoglobulin receptor (KIR) variants exhibit balancing selection to modulate placental invasion versus immune rejection (Armstrong et al., 2017). Similarly, co-opted viral elements in the genome, such as, syncytin genes from endogenous retroviruses, have been positively selected for their role in placental development (Dunn-Fletcher et al., 2018). An example of gene co-option is the integration of an anthropoid-specific retroviral enhancer upstream of the corticotropin-releasing hormone (*CRH*) gene. This regulatory element (THE1B) boosts placental *CRH* expression in humans and apes. Introduced into mice as a transgene, the human *CRH* locus significantly altered gestation length in mice (Dunn-Fletcher et al., 2018), demonstrating how evolutionary novelties in gene regulation can directly affect birth timing. Such findings reinforce that human pregnancy has been shaped by unique selective pressures, from anatomical constraints to immune challenges, distinguishing our birth timing mechanisms from those of other mammals.

Within human populations, genomic scans for selection have revealed a mosaic of evolutionary forces acting on PTB-related loci. Complex traits like spontaneous PTB do not follow a single story of natural selection; instead, different genetic regions show evidence of unique evolutionary histories. LaBella et al. (2020) applied a suite of population genetics tests to known PTB-associated genomic regions, identified via GWAS, and found enrichment of multiple signatures. Some loci bore marks of purifying (negative) selection or conservation, while others showed high population differentiation, suggesting local adaptation, and yet others had signals of accelerated evolution or balancing selection (LaBella et al., 2020). Strikingly, 120 out of 215 PTB-linked regions had significant evidence of at least one type of selection, exceeding what random genomic regions would show (LaBella et al., 2020). These results indicate that no single evolutionary force is responsible for PTB genetics, rather, human birth timing has been shaped by a mosaic of evolutionary forces, likely reflecting trade-offs and varying selective pressures in different environments (LaBella et al., 2020). For instance, alleles that protect against infection-triggered PTB might rise in one population (positive selection), while variants that mediate maternal-fetal resource allocation might be maintained at intermediate frequencies globally.

Another example of population-level adaptation is the progesterone receptor gene (PGR), which is critical for maintaining pregnancy. As described above, comparative genomics reveals that *PGR* experienced adaptive evolution on the human lineage. When comparing mammals, *PGR* has an excess of amino acid replacements specifically in humans and chimpanzees, a hallmark of positive selection on its protein function (Li et al., 2018). Those human-specific changes cluster in the receptor’s regulatory domains, hinting at a rewiring of progesterone signaling that may underpin the unique mechanism of human parturition (Li et al., 2018). A study showed that natural selection has differentiated the PGR allele pool among human groups as East Asian populations underwent a very recent selective sweep at the PGR locus, nearly fixing certain derived alleles, whereas European populations retain a highly polymorphic *PGR* haplotype structure suggestive of long-term balancing selection (Li et al., 2018). These evolutionary differences have meaningful biological impacts. In East Asian populations, the selected *PGR* variants change how much of the protein is produced (particularly in ovaries) and offer measurable protection against early spontaneous preterm birth selection (Li et al., 2018). European populations, meanwhile, maintain greater PGR diversity, possibly reflecting a balancing act where different variants optimize different aspects of reproduction or survival rather than pregnancy length alone selection (Li et al., 2018). Interestingly, ancient DNA reveals that Neanderthals carried a PGR variant that no longer exists in pure form, but when this variant was passed to modern humans through ancient interbreeding, it increased preterm birth risk and other health issues (Zeberg et al., 2020). PGR thus illustrates how evolution can tinker with a single gene to create population-specific flavors of the same pregnancy pathway. These genetic differences likely help explain why preterm birth rates vary between ethnic groups. e.g., why Asian populations often show different rates than African or European ancestry populations. (Li et al., 2018). Evolutionary analyses not only retrace this history but also spotlight genetic loci that modern biomedical research can target to understand and perhaps mitigate the risk of preterm birth (Plunkett et al., 2011).

## Genome editing technologies applied to PTB research

Genetic manipulation approaches used to study preterm birth (PTB) span multiple biological scales and are often discussed collectively despite operating through fundamentally distinct mechanisms. CRISPR/Cas9 represents a targeted genome-editing technology that directly modifies endogenous DNA sequences through programmable nuclease activity, enabling precise loss-, gain-, or alteration-of-function at specific loci (Jinek et al., 2012; Doudna and Charpentier, 2014). In contrast, Bacterial Artificial Chromosome (BAC) transgenesis introduces large exogenous genomic fragments, frequently encompassing complete genes and their regulatory landscapes, allowing interrogation of complex, developmentally regulated, and human-specific expression programs that are difficult to model through minimal sequence edits alone ((Dunn-Fletcher et al., 2018). Suppressor tRNA–based strategies operate at a different level altogether, functioning at the translational stage by bypassing premature stop codons to restore protein expression without altering genomic DNA, and thus represent a form of conditional genetic suppression rather than genome editing (Beier and Grimm, 2001). Together, these complementary approaches provide distinct yet synergistic tools for dissecting the genetic, regulatory, and functional architecture of PTB across cellular, organismal, and evolutionary contexts.

## CRISPR/Cas9 genome editing in PTB models

Clustered Regularly Interspaced Short Palindromic Repeats/CRISPR-associated protein 9 (CRISPR/Cas9) has revolutionized functional genomics with its efficiency and versatility (Balasubramanian et al., 2024). The CRISPR/Cas9 system uses a short guide RNA (gRNA) to target a specific DNA sequence, where the Cas9 endonuclease creates a double-strand break (Kalebic et al., 2016). The cell’s innate DNA repair pathways then act. In the absence of a repair template, non-homologous end joining (NHEJ) often introduces small insertions/deletions that disrupt the gene (knockout). In contrast, if a donor template is provided, homology-directed repair (HDR) can precisely edit or insert sequences (knock-in) (Nakamura et al., 2023). This mechanism, a targeted cut followed by error-prone repair, makes CRISPR a powerful tool to generate loss-of-function mutations or specific genetic alterations at will. CRISPR/Cas9 enables scientists to manipulate genes in trophoblast cell lines, organoids, or animal models to see how these changes affect pregnancy outcomes (Mao et al., 2023; Dong et al., 2022).

One key application is creating placental cell models with gene knockouts/knockins to study cellular functions relevant to PTB. For instance, Guerrero-Santoro, Morizane (Guerrero-Santoro et al., 2023) used CRISPR/Cas9 to knock out the *PNPLA9* gene in a human trophoblast cell line (BeWo) to investigate placental lipid metabolism. The engineered *PNPLA9*-null trophoblasts showed altered lipid droplet accumulation, revealing *PNPLA9*’s role in placental lipid turnover. By analyzing such phenotypes, researchers can link gene function to conditions like fetal growth restriction or inflammation that are risk factors for PTB. CRISPR has also been employed in primary trophoblast stem cells and placental organoids, which better mimic the *in vivo* placenta. Notably, CRISPR allowed the first stable gene-edited human placental organoids, with the production of *ACE2*-knockout organoids (Arthurs et al., 2025). In that study, they also introduced a specific SNP (rs2074192) into the *ACE2* gene in organoids, creating isogenic models to examine how a human genetic variant influences placental biology (Arthurs et al., 2025). These placental organoid models, essentially mini-placentas grown in culture provide a novel system to study infection, immunity, and drug responses related to PTB. Beyond interrogating specific variants, CRISPR/Cas9 enables systematic functional validation of preterm birth candidate genes. If human genetic studies implicate a particular gene, researchers can use CRISPR to knock it out in relevant cell types or introduce risk alleles to directly test effects on key processes such as progesterone signaling or inflammatory cytokine production. The technology scales efficiently to genome-wide screens through high-throughput approaches.

In animal models, CRISPR is expediting the creation of mouse models of PTB. Traditional knockouts often took years to develop, but with CRISPR, researchers can rapidly generate mice lacking specific genes to see if they exhibit pregnancy phenotypes (preterm labor, fetal loss, or developmental abnormalities). For example, knocking out genes involved in cervical remodeling or uterine quiescence in mice can induce premature labor, modeling aspects of human PTB. CRISPR can also introduce human-specific mutations into mice (humanized models). This was elegantly shown with the *CRH* gene, while mice do not naturally express placental *CRH* like humans do, CRISPR engineering has been used to insert the human *CRH* regulatory sequence into mice, affecting gestation timing (Dunn-Fletcher et al., 2018). Beyond *CRH*, CRISPR-based genome editing is increasingly used to interrogate additional pathways implicated in preterm birth. For example, disruption of uterine *Trp53* signaling results in premature uterine senescence and spontaneous preterm labor, highlighting a role for cellular stress responses in maintaining pregnancy (Hirota et al., 2010), while loss of extracellular matrix components such as biglycan and decorin leads to cervical and fetal membrane defects and early delivery, modeling structural contributors to PTB (Calmus et al., 2011). Furthermore, *in utero* genome editing is an emerging approach in which CRISPR components are delivered to the fetus or placenta during gestation to correct lethal developmental disorders. Although still experimental, this strategy points out the potential for prenatal genetic interventions to mitigate perinatal complications, including those that predispose to PTB or pregnancy loss. Despite this promise, we must remain cautious because alteration of a gene may have profound effects (e.g., pleitropy), regardless of its preferential expression or of evolutionary origin.

## BAC transgenesis and large DNA insertions

While CRISPR excels at small edits, Bacterial Artificial Chromosome (BAC) transgenesis enables the introduction of large segments of DNA (tens to hundreds of kilobases) into model organisms. A BAC is a large circular DNA construct that can carry entire genes along with their native regulatory elements, which are promoters, enhancers, and introns (Dewar et al., 2022). BAC transgenesis involves injecting these constructs into zygotes or embryonic stem cells to create transgenic animals that carry the introduced DNA, often randomly integrated into the genome. The main advantage of BACs is that they preserve the complex regulation of a gene, something crucial for genes with tissue-specific or developmental stage-specific expression that a minimal cDNA transgene might not recapitulate (Mogi et al., 2025). In PTB research, BAC transgenesis is particularly valuable for studying human-specific genes or regulatory sequences that have no analog in common animal models such as mice. By inserting a human BAC into mice, one can humanize certain aspects of pregnancy in the mouse and observe the outcome on gestational timing or pregnancy maintenance (Dong et al., 2024).

A striking example involves the corticotropin-releasing hormone gene (CRH), which is produced by the human placenta and thought to be a part of the placental clock regulating birth timing. Rodents lack placental CRH expression, so to test its role, researchers created mice transgenic for a human CRH BAC that included an anthropoid primate-specific enhancer (THE1B retroviral element). The result was that these BAC-transgenic mice showed significantly prolonged gestation lengths compared to wild-type mice (Dunn-Fletcher et al., 2018). In effect, introducing the human regulatory DNA into the mouse placenta altered the timing of birth, providing functional evidence that the human-specific evolution of CRH regulation can influence pregnancy duration. This BAC approach thus allowed scientists to model a uniquely human facet of pregnancy (CRH dynamics) in an animal and directly link it to parturition timing (Dunn-Fletcher et al., 2018).

BAC insertion is also useful for rescuing lethal phenotypes and studying gene function specifically in the placenta or reproductive tissues. Many genes that affect pregnancy (such as those regulating placental development or embryo implantation) are essential for embryonic development, so a conventional knockout is embryonically lethal and does not allow one to study later pregnancy stages. In such cases, a BAC carrying a tissue-specific expression of the gene can be introduced. For example, in the case of the lipid droplet regulator CGI-58 (*Abdh5*), global knockout in mice causes early lethality, but researchers created a placenta-specific rescue by adding back CGI-58 expression only in trophoblast cells (Guerrero-Santoro et al., 2023). This selective restoration in the placenta reversed the placental lipid accumulation phenotype of the knockout (Guerrero-Santoro et al., 2023), proving that the observed placental dysfunction (and likely fetal growth issues) were due to the lack of CGI-58 in the placenta. Although that study used a conditional genetic strategy rather than a BAC, it illustrates the principle: one can supply a gene to a specific tissue (via a BAC with a trophoblast-specific promoter, or via viral delivery to the placenta) to test the sufficiency of that gene in preventing PTB-related pathology. BAC transgenes have the capacity to include all regulatory elements for correct spatial expression, which is often critical in reproductive biology, where gene expression must be tightly controlled in time and place.

Finally, BAC and related large-fragment methods contribute to modeling complex loci associated with PTB. Some GWAS-identified risk regions span gene clusters or non-coding regulatory landscapes. By engineering a BAC that encompasses the entire risk haplotype (for instance, a cluster of cytokine genes or hormone receptors) and introducing it into an animal, researchers can observe the combined effect of those variants on pregnancy outcome. This is particularly useful if multiple linked variants jointly influence PTB risk.

## Suppressor tRNA approaches for genetic suppression

In the realm of PTB research, suppressor tRNA strategies are still emerging, but they hold promise for tackling certain genetic contributors to preterm birth. For example, consider a scenario where a particular gene essential for maintaining uterine quiescence or fetal membrane integrity has a known nonsense mutation in some patients that predisposes to PTB. Traditional gene editing to fix that mutation in every cell of the patient is challenging, but a suppressor tRNA delivered to the uterus or placenta might allow the patient’s cells to ignore the mutation’s stop signal, restoring the missing protein function during pregnancy. This could be envisioned as a gene therapy for monogenic causes of PTB. While such therapy is hypothetical at this stage, preclinical studies in other fields are paving the way–showing that installing a suppressor tRNA can rescue diseases like cystic fibrosis and muscular dystrophy in cell and animal models (Pierce et al., 2025). The placenta could be an accessible target for tRNA therapy via intrauterine injection or nanoparticle delivery, given its compartmentalized nature.

In research applications, suppressor tRNAs can be used to create conditional knockdown models. One clever approach is to engineer an animal or cell line so that a vital pregnancy gene is replaced by a version containing an early stop codon; this renders the gene nonfunctional unless a suppressor tRNA is present. By controlling the expression of the suppressor tRNA (for instance, making it inducible by a drug or expressing it only in certain tissues), researchers gain a switch to turn the gene’s function “on” or “off” at will. This method can achieve a level of temporal control that standard knockouts lack–crucial for pregnancy genes that must function at specific gestational windows. For example, a gene might be required early in pregnancy for placental development but later its expression must taper off for labor to initiate. A suppressor tRNA system could potentially mimic this by withdrawing the suppressor at late gestation to naturally terminate the gene’s expression via the engineered stop codon. Though these experimental setups are complex, they offer a precise tool to dissect timing in reproductive biology.

Beyond endogenous genes, suppressor tRNAs intersect with expanding the genetic code–scientists have used them to insert unnatural amino acids into proteins, which could be used to tag or modulate proteins involved in parturition. For instance, a suppressor tRNA system could incorporate a photoactivatable amino acid into a uterine contraction protein, allowing researchers to trigger or halt its function with light and study immediate effects on uterine tissue contractility. It is worth noting that suppressor tRNA technology must be applied cautiously, as global suppression of stop codons can be deleterious (cells have many essential stop codons). Thus, current strategies often involve targeted suppressor tRNAs that only recognize a unique stop codon context or are coupled with selective delivery. The prime-edited suppressor tRNA approach is disease-agnostic and elegant in this regard: by converting a naturally occurring tRNA gene to a suppressor, it leverages the cell’s own transcriptional regulation of tRNAs and specifically addresses one mutation of interest (Pierce et al., 2025). In the context of PTB, this could mean editing a woman’s cells to carry a tRNA that suppresses, say, a premature stop in the *COL6A1* gene (if a collagen defect were causing fetal membrane weakness). The edited tRNA would only be active where that specific stop mutation’s mRNA is present, minimizing off-target effects. Such precision medicine approaches, although in early stages, exemplify the innovative genetic tools being considered to prevent PTB.

## FDA-approved base-editing or gene-editing-related therapeutic cases

Although gene editing for pregnancy-related conditions has not yet reached clinical practice, recent regulatory approvals in other fields provide useful insights. In December 2023, the U.S. Food and Drug Administration approved *Casgevy*, a CRISPR-based therapy for sickle cell disease. This approval highlighted several regulatory requirements, including extensive off-target analysis, long-term follow-up of treated patients, and strict manufacturing standards (Philippidis, 2024). Similarly, the investigational therapy *VERVE-101*, designed for hereditary transthyretin amyloidosis, represents an early example of *in vivo* base editing entering clinical testing. Its development points out the importance of biodistribution studies and tissue-specific safety evaluation (Hooper et al., 2024).

However, regulatory guidance from the FDA further emphasizes careful risk–benefit assessment when developing therapies for pregnant individuals (David et al., 2022). This is because current interventions for preterm birth remain limited, as gene editing strategies may eventually qualify for accelerated regulatory pathways, although long-term monitoring and strict manufacturing standards will likely be required. Any future gene editing therapy for preterm birth would likely be used alongside existing interventions rather than replacing them entirely.

## Gene-editing delivery strategies

For gene-editing approaches to become clinically useful for preventing preterm birth (PTB), one of the most important challenges is delivering editing tools precisely to uterine tissues while limiting unintended distribution throughout the body. This is because uterine tissues are accessible by highly local routes (transcervical/intrauterine and intravaginal), yet are richly vascularized and lymphatically connected, enabling unintended systemic exposure even after local dosing (Red-Horse, 2008). However, some delivery strategies, including virus-based platforms such as virus-like particles (VLPs), lipid and polymeric nanoparticle systems, and localized delivery approaches, have been designed to concentrate therapeutic agents in uterine tissues improving tissue specificity while reducing systemic exposure (Red-Horse, 2008; Charlton Hume et al., 2019).

Virus-like particles (VLPs) mimic viruses in their ability to enter cells but do not contain genetic material, meaning they cannot replicate or cause infection. Due to this ability, they combine efficient delivery with a favorable safety profile (Charlton Hume et al., 2019). Importantly, VLPs can be engineered to carry molecules that recognize receptors found in uterine tissues. For example, targeting ligands that bind to receptors such as integrin α5β1 may help guide these particles specifically to gestational tissues (Nooraei et al., 2021). Furthermore, Lipid nanoparticles (LNPs), similar to those used in mRNA vaccines, can be modified with peptides that recognize pregnancy-related proteins such as pregnancy-associated plasma protein-A (PAPP-A), which may help direct therapeutic molecules toward reproductive tissues (Mitchell et al., 2021). Other materials, such as polymeric PLGA nanoparticles, allow controlled release of therapeutic cargo and have already been widely studied for safety in pregnancy-related applications (Keelan et al., 2015; Yang and Wang, 2024).

These approaches could be optimized using passive targeting, which relies on physiological characteristics of tissues, including enhanced vascular permeability, while active targeting uses ligands that bind to specific cell receptors (Yu et al., 2010). *In vivo* studies show that using RGD ligands attached to lipid nanoparticles (LNPs) can increase gene expression in the endometrium while reducing off-target accumulation in organs such as the liver and spleen (Abbasi et al., 2026). Similarly, receptor-targeted liposomes designed to bind the oxytocin receptor in the pregnant myometrium have demonstrated enhanced uterine drug delivery (Refuerzo et al., 2016). In animal models, oxytocin receptor–targeted liposomes doubled uterine drug concentrations while reducing fetal drug exposure by limiting placental transfer (Refuerzo et al., 2016). Furthermore, these approaches could be delivered via local administration routes, including intrauterine, intracervical, or transvaginal delivery, which may help concentrate treatment in reproductive tissues while minimizing systemic exposure (Zhang et al., 2019).

Despite the introduction and advancement of these delivery strategies, there is a growing concern about whether therapeutic molecules could cross the placenta and reach fetal tissues. The placenta provides an important protective barrier, and the likelihood of transfer depends on factors such as molecular size, electrical charge, and the type of delivery vehicle used (Yang et al., 2012). Larger nanoparticles, particularly those greater than 50 nm in diameter, appear less likely to cross the placental barrier. Similarly, CRISPR-Cas9 ribonucleoprotein complexes are relatively large molecules and may be inherently limited in their ability to enter fetal circulation (Muoth et al., 2016; van Kammen et al., 2025). However, some viral vectors have shown variable levels of placental transfer, with certain adeno-associated virus (AAV) serotypes demonstrating higher rates than others (Nakamura et al., 2019). Therefore, designing delivery vehicles large enough to limit placental passage, targeting molecules specifically to maternal tissues, restricting interventions to certain stages of pregnancy, and potentially using pharmacological approaches that strengthen placental barrier function could help mitigate this effect (Pepe and Albrecht, 2021).

### A blueprint for identifying candidate protein coding genes for editing approaches to PTB

**FIGURE 2:**
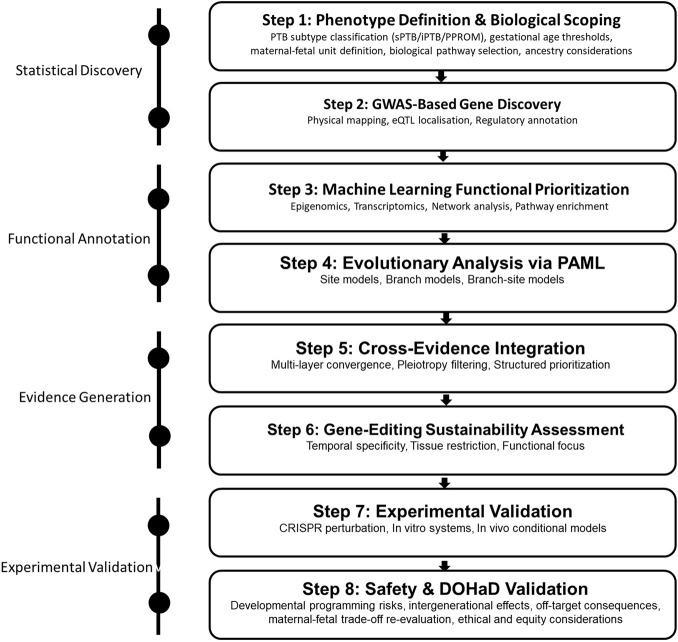
Proposed Methods for genome editing in pregnancy. An eight-step framework for systematically identifying and validating candidate genes for therapeutic intervention in preterm birth. The workflow progresses from statistical discovery (phenotype definition and GWAS mapping) through functional annotation (machine learning and evolutionary analysis) to evidence integration and prioritization.

The proposed framework consists of eight integrated steps, each designed to refine candidate gene lists through progressively stringent biological and safety filters (Figure 2). Beginning with GWAS discovery, the pipeline incorporates functional genomic annotations, machine learning-based prioritization, phylogenetic selection analyses, and explicit evaluation of developmental safety. The approach is inherently conservative, prioritizing genes with convergent support from multiple independent lines of evidence while explicitly excluding those with extensive pleiotropy or essential developmental roles. Although focused on maternal genetic contributions to spontaneous PTB, the general architecture is adaptable to fetal genetics and related pregnancy complications.

## Methods for genome editing in pregnancy

An eight-step framework for systematically identifying and validating candidate genes for therapeutic intervention in preterm birth. The workflow progresses from statistical discovery (phenotype definition and GWAS mapping) through functional annotation (machine learning and evolutionary analysis) to evidence integration and prioritization.

### Step 1: phenotypic and biological scope definition

Rigorous candidate identification begins with precise phenotype definition. PTB phenotype must be explicitly defined (spontaneous labor, PPROM, or combined), excluding medically indicated deliveries, to ensure etiological homogeneity. The maternal genome is prioritized as it directly regulates the uterine, cervical, and decidual processes initiating labor, though fetal contributions merit parallel consideration. Crucially, biological scope could be constrained to genes functioning in pathways directly governing parturition initiation: inflammatory signaling (cytokine cascades, NFκB activation), endocrine regulation (progesterone/estrogen signaling, prostaglandin synthesis), myometrial contractility (gap junction formation, calcium dynamics), cervical remodeling (extracellular matrix turnover), and placental endocrine signaling (CRH production, glucocorticoid metabolism) (Mead et al., 2023). This constraint minimizes inclusion of genes acting through indirect, pleiotropic, or developmentally essential mechanisms unsuitable for targeted intervention.

### Step 2: GWAS-based discovery

Rather than restricting analyses to genome-wide significant loci, the proposed framework would retain both significant and sub-threshold associations to capture PTB’s polygenic architecture. Single nucleotide polymorphisms would be mapped to genes through physical proximity, expression quantitative trait locus (eQTL) colocalization in pregnancy-relevant tissues (decidua, myometrium, cervix, placenta), and regulatory annotations based on chromatin state and transcription factor binding disruption. This comprehensive mapping would generate a GWAS-anchored gene universe that serves as input for functional refinement, balancing statistical rigor with biological completeness.

### Step 3: machine learning-based functional prioritization

To distinguish causal genes from statistical noise, machine learning models would integrate multi-dimensional functional features: tissue-specific epigenomic activity (chromatin accessibility, histone modifications), transcriptional profiles in pregnancy tissues, gestational age-dependent expression trajectories, protein-protein interaction networks, and regulatory effect predictions. Recent foundation models for biological sequences, including ProteinBERT, ESM-3, Enformer, and Geneformer, provide a powerful framework for integrating sequence-level, regulatory, and functional information and can be fine-tuned on pregnancy- and PTB-relevant datasets to identify genes contributing to preterm birth (PTB), as well as to prioritize functional noncoding regulatory elements (Theodoris et al., 2023; Avsec et al., 2021; Hayes et al., 2025; Rives et al., 2021; Brandes et al., 2022). Recent advances in machine learning have proven particularly powerful for integrating comparative genomics with functional profiling, enabling systematic cataloging of cross-species differences in gene expression and cis-regulatory element (CRE) function (Gilbertson and Reilly, 2025). These approaches can leverage evolutionary conservation patterns alongside species-specific regulatory innovations to refine gene prioritization. Furthermore, AI-driven predictive models have demonstrated success in CRISPR/Cas9 target prediction and off-target effect minimization (Patel et al., 2025), suggesting that similar deep learning frameworks could be applied to optimize functional validation strategies and to filter likely non-functional coding and noncoding candidates among PTB-associated loci. Models would be trained on curated sets of known PTB-associated genes with functional validation, optimizing for high precision to minimize false positives. By incorporating evolutionary constraint scores, tissue-specific regulatory annotations, and cross-species functional genomic data, machine learning classifiers can distinguish genes with authentic regulatory roles in parturition from those showing spurious GWAS associations. Genes demonstrating regulatory activity specifically in decidua, myometrium, or cervix, particularly within inflammatory or hormonal pathways, would receive elevated priority scores. This step would refine the GWAS-anchored set by emphasizing genes with coherent biological roles in parturition timing rather than peripheral or ubiquitous functions.

### Step 4: evolutionary context through PAML analysis

Evolutionary analysis would provide orthogonal evidence regarding gene function and constraint. Using phylogenetic models implemented in PAML (Phylogenetic Analysis by Maximum Likelihood), candidate genes would be evaluated for selection signatures across placental mammals (Yang, 2007). Three complementary models would be applied: site models would identify codons under recurrent positive or purifying selection; branch models would test for lineage-specific shifts in selective pressure along primate or human lineages; and branch-site models would detect episodic positive selection at specific residues in defined lineages. Genes under strong purifying selection would be interpreted as core regulators of pregnancy maintenance, their conservation across species suggests functional criticality. Conversely, genes exhibiting lineage-specific positive selection may reflect adaptive tuning of gestational timing or placentation strategies, potentially indicating species-specific regulatory elements. Both evolutionary signatures would provide biological insight, though they may suggest different therapeutic considerations: conserved genes represent robust but potentially less flexible targets, while adaptively evolving genes may offer human-specific intervention opportunities but require careful safety evaluation.

### Step 5: cross-evidence integration and prioritization

Evidence from GWAS, functional genomics, and evolutionary analyses would be synthesized using a structured decision matrix. Highest-priority candidates would demonstrate convergent support: statistical association with PTB, functional activity in pregnancy-relevant tissues, membership in parturition-related pathways, and non-neutral evolutionary signatures. Genes would be explicitly deprioritized if they exhibit broad developmental expression, extensive pleiotropy across multiple organ systems, or early embryonic lethality in model organisms. This integration step would distinguish genes with pathway-specific, late-gestation roles from those with systemic or developmentally essential functions, thereby focusing on targets amenable to selective intervention with minimal off-target effects.

### Step 6: gene-editing suitability assessment

From the integrated candidate list, genes would be evaluated for practical gene-editing feasibility. Favorable characteristics would include: predominantly regulatory effects rather than structural roles, temporal expression patterns restricted to late gestation, tissue-specific activity in uterus or cervix, and limited systemic expression. Genes with widespread tissue distribution, essential housekeeping functions, or documented roles in non-pregnancy physiology would be flagged for additional scrutiny. This assessment would ensure that prioritized candidates are not only biologically relevant but also practically targetable through gene-editing approaches with acceptable specificity.

### Step 7: experimental validation framework

High-priority candidates would require functional validation before consideration for therapeutic development. Validation would employ CRISPR-based perturbation in pregnancy-relevant cellular systems, primary myometrial cells, decidual stromal cells, and trophoblast models, to assess effects on inflammatory signaling, hormonal responsiveness, and contractility. *In vivo* validation would utilize conditional or tissue-specific gene perturbation in mouse models with phenotypic characterization of gestational length, labor initiation timing, and pathway-specific readouts. These experiments would establish causality and mechanism, distinguishing true regulators of parturition from genes whose association reflects linkage disequilibrium or indirect effects. Importantly, this validation framework would be designed for biological insight rather than immediate therapeutic application, maintaining focus on mechanistic understanding.

### Step 8: developmental safety and DOHaD considerations

All candidate genes undergo explicit safety evaluation through a Developmental Origins of Health and Disease (DOHaD) lens. This assessment considers evolutionary pleiotropy (genes with functions in multiple physiological systems), associations with adult disease phenotypes in GWAS databases, temporal expression during fetal development, and potential for maternal-fetal trade-offs where maternal benefit might compromise offspring health. Genes would be classified not only by predicted efficacy but also by long-term safety profiles, ensuring that any future therapeutic development incorporates intergenerational health considerations from the outset.

Women born preterm have approximately 40% higher odds of delivering their own babies preterm, suggesting intergenerational transmission (Boivin et al., 2012). While CRISPR and related technologies have sparked interest in editing embryonic genomes to eliminate PTB risk factors, such interventions carry substantial risks. Single-cell analyses of CRISPR-edited early human embryos reveal off-target mutations, large on-target deletions/rearrangements, loss of heterozygosity beyond target loci, and segmental aneuploidy, genomic alterations that standard genotyping may miss (Kosicki et al., 2018; Alanis-Lobato et al., 2021). These changes could compromise health, increase cancer risk through p53-mediated selection of damage-resistant cells (Haapaniemi et al., 2018), and would be inherited by future generations (Rubeis and Steger, 2018).

Moreover, many genes involved in parturition exhibit pleiotropy, the CCR5 case exemplifies how editing for one trait (HIV resistance) can produce unintended system-wide effects on immunity and cognition (Meyer, 2020). While moderate evidence from animals and humans demonstrates that gene alterations can have multisystem consequences, we lack specific data on PTB-related genes. Critically, DOHaD-relevant epigenetic mechanisms, particularly imprinting, can transmit developmental effects from germline perturbations to offspring in mammalian models, even when the intended edit is achieved (Horii et al., 2025). This underscores that interventions targeting reproductive biology carry unique ethical and biological responsibilities given their potential transgenerational effects.

## Limitation of knowledge available

The genetic association evidence synthesized is predominantly from GWAS and candidate gene studies conducted in populations of European and East Asian ancestry, a well-documented bias with particular relevance to PTB research. African-ancestry populations, who bear the highest global burden of preterm birth, remain severely underrepresented in the GWAS literature, meaning that effect sizes and risk allele frequencies reported in Table 1 may not generalize across populations (Ojewunmi and Fatumo, 2025). Population-specific evolutionary histories, including distinct linkage disequilibrium patterns and allele frequencies, further limit the transferability of findings between ancestry groups. Additionally, the evolutionary signatures reviewed (positive selection, gene duplication, and transposable element co-option) do not establish causality between evolutionary change and contemporary PTB risk, and for several loci in Table 1 the association evidence remains preliminary or unreplicated. The proposed gene-editing framework is conceptual rather than empirically validated, and the heterogeneity of PTB as a syndrome means no single gene or pathway is likely to account for substantial risk across populations. Larger, ancestrally diverse, and phenotypically well-characterized cohort studies are needed to advance the field.

## Conclusion

This review demonstrates that evolutionary biology provides essential context for understanding and addressing preterm birth. Human pregnancy reflects competing selective pressures, from metabolic constraints, pelvic anatomy, immune tolerance, and ecological timing, all factors which have shaped genes controlling birth timing. Population-specific evolutionary histories, including recent selection on *PGR* and adaptive evolution of immune genes, explain ethnic disparities in PTB risk and identify therapeutic targets. Comparative vertebrate analyses reveal that gestation length is highly evolvable and shaped by multiple ecological factors. Understanding which aspects of human pregnancy are deeply conserved versus recently evolved clarifies which genomic regions can be safely targeted for intervention. The mosaic of evolutionary forces acting on PTB-associated loci reflects fundamental reproductive trade-offs. Modern genome editing technologies, CRISPR/Cas9, BAC transgenesis, and emerging approaches, enable functional validation and therapeutic development. However, evolutionary analysis provides critical guidance: rapidly evolving, population-specific genes represent safer therapeutic targets than highly conserved genes with pleiotropic effects.
